# Hypocretin/Orexin Neuropeptides: Participation in the Control of Sleep-Wakefulness Cycle and Energy Homeostasis

**DOI:** 10.2174/157015909787602797

**Published:** 2009-03

**Authors:** A Nuñez, M.L Rodrigo-Angulo, I. De Andrés, M Garzón

**Affiliations:** Departamento de Anatomía, Histología y Neurociencia, Facultad de Medicina, Universidad Autónoma de Madrid, Madrid, Spain

**Keywords:** Posterior lateral hypothalamic area, hypocretin neurons, orexin neurons, perifornical area, sleep-wakefulness, food intake.

## Abstract

Hypocretins or orexins (Hcrt/Orx) are hypothalamic neuropeptides that are synthesized by neurons located mainly in the perifornical area of the posterolateral hypothalamus. These hypothalamic neurons are the origin of an extensive and divergent projection system innervating numerous structures of the central nervous system. In recent years it has become clear that these neuropeptides are involved in the regulation of many organic functions, such as feeding, thermoregulation and neuroendocrine and cardiovascular control, as well as in the control of the sleep-wakefulness cycle. In this respect, Hcrt/Orx activate two subtypes of G protein-coupled receptors (Hcrt/Orx1R and Hcrt/Orx2R) that show a partly segregated and prominent distribution in neural structures involved in sleep-wakefulness regulation. Wakefulness-enhancing and/or sleep-suppressing actions of Hcrt/Orx have been reported in specific areas of the brainstem. Moreover, presently there are animal models of human narcolepsy consisting in modifications of Hcrt/Orx receptors or absence of these peptides. This strongly suggests that narcolepsy is the direct consequence of a hypofunction of the Hcrt/Orx system, which is most likely due to Hcrt/Orx neurons degeneration.

The main focus of this review is to update and illustrate the available data on the actions of Hcrt/Orx neuropeptides with special interest in their participation in the control of the sleep-wakefulness cycle and the regulation of energy homeostasis. Current pharmacological treatment of narcolepsy is also discussed.

## INTRODUCTION

Hypocretins/orexins (Hcrt/Orx) are hypothalamic neuropeptides that are synthesized by neurons located mainly in the perifornical area of the posterolateral hypothalamus. These hypothalamic neurons are the origin of an extensive and divergent projection system innervating numerous structures of the central nervous system (CNS). Hcrt/Orx neuropeptides are involved in the regulation of many organic functions, such as feeding, thermoregulation and neuroendocrine and cardiovascular control, as well as in the control of the sleep-wakefulness cycle and expression of narcolepsy. Since the discovery of the Hcrt/Orx neuropeptides in 1998 much information has been gathered about their actions in enhancing wakefulness and EEG activation. As well as increasing wakefulness and food intake, administration of Hcrt/Orx neuropeptides also affects blood pressure, hormone secretion and locomotor activity (see for recent review [19]).

## HYPOCRETINS/OREXINS

**Fig. (1):**
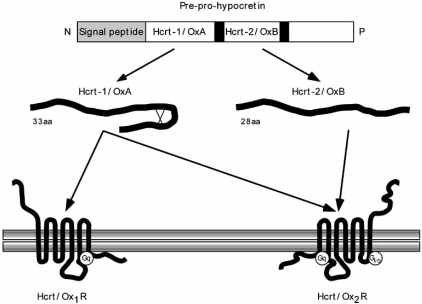
Schematic depiction of hypocretin/orexin system. Hypocretin-1/Orexin A (Hcrt-1/OrxA) and hypocretin-2/Orexin B (Hcrt-2/OrxB) are derived from a common precursor peptide, pre-pro-hypocretin. After removal of the N-terminal secretory signal sequence, pre-prohypocretin is cleaved at specific sites having basic amino acid residues to yield the two mature peptides. Hcrt-1/OrxA possesses two disulfide bridges while Hcrt-2/OxB is linear. The actions of hypocretins are mediated through interaction with two heterotrimeric G protein-coupled receptors (Hcrt/Orx_1_R and Hcrt/Orx_2_R), whose distribution in the central nervous system is regionally specific. Hcrt/Orx_1_R is more selective for Hcrt-1/OrxA, while Hcrt/Orx_2_R is equally specific for both peptides. Hcrt-1/OrxA is linked exclusively to excitatory G proteins of the Gq subclass, whereas Hcrt-2/OxB couples *in vitro* to excitatory Gq and/or inhibitory Gi/o. Signaling through Gq pathway results in increase of intracellular Ca^2+^, most probably *via* activation of phospholipase C-b with subsequent triggering of the phosphatidylinositol cascade and activation of protein kinase C. The Ca^2+^ influx likely induces depolarization. Signaling via inhibitory Gi/o pathway may occur through hyperpolarization due to K+ efflux (GIRK channel-mediated). Figure modified from [9].

Two independent research groups (the De Lecea and Sakurai groups) simultaneously described the existence of two peptides synthesized by hypothalamic neurons [18,75]. De Lecea and collaborators observed that these peptides are expressed by neurons in the posterolateral hypothalamus that are very similar to the secretin-related peptides, so they named them hypocretin-1 and hypocretin-2 (Hcrt-1 and Hcrt-2; [18]). At the same time, Sakurai *et al*. [74,75] reported that central administration of these peptides increased feeding behavior and called them orexin A (OrxA) and orexin B (OrxB). Hcrt/Orx neuropeptides act on two types of receptors (ORX1R and ORX2R; also known as Hcrtr1R and Hcrtr2R; [75]), which are expressed throughout the CNS (Fig. **1**).

Mammalian Hcrt/Orx1 is a 33 amino acid peptide with a molecular mass of roughly 3.5 kDa; it possesses an N-terminal pyroglutamyl residue, a C-terminal amidation, and two intramolecular disulfide bridges, Cys6–Cys12 and Cys7-Cys14. The amino acid sequence of Hcrt/Orx1 is remarkably well preserved in humans, cattle, rats, mice [75], and pigs [20].

Mammalian Hcrt/Orx-2 is a 28 amino acid peptide with a molecular mass of about 2.9 kDa and a C-terminal amidation. The structure of Hcrt/Orx-2 in solution has been determined by magnetic resonance imaging [47], and consists of two stable alpha-helices connected by a short linker. It shows 46% (13/28) amino acid identity to Hcrt/Orx1. Rat and mouse Hcrt/Orx-2 are identical, and only one and two amino acid residues are changed in the porcine and human counterparts, respectively. Hcrt/Orx neuropeptides that have also been described in the frog *Xenopus laevis* has a high similarity to the mammalian peptides [78]. The structure of Hcrt/Orx belongs to the incretin family of neuropeptides and has been strongly conserved during the evolution.

The Hcrt/Orx gene is located in chromosome 17q21-q24 [76]. In humans this gene consists of two exons and one intron, and encodes a 131 amino acid precursor peptide, prepro-Hcrt/Orx. This precursor possesses an N-terminal 33 residue secretory signal peptide, and is cleaved at sites of basic amino acid residue pairs by prohormone convertases to yield Hcrt/Orx1 and Hcrt/Orx2 [76]. The amino acid identity between human and rat prepro-Hcrt/Orx is 83%, with most substitutions occurring near the C-terminus. Given this structure, the existence of a third functional peptide derived from the C-terminal part of the precursor is unlikely.

**Fig. (2):**
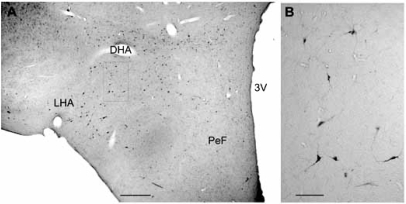
Distribution of Hcrt/Orx neurons in the cat hypothalamus. A: Microphotograph of a coronal section of cat hypothalamus showing the distribution of orexinergic neurons as result of the immunoreaction for anti-Orexin A antiserum. No counterstaining. B: High magnification of area squared in A. DHA. dorsal hypothalamic area, LHA: lateral hypothalamic area, PeF: perifornical region, 3V: third ventricle. Calibration bars: A, 500 µm, B, 100 µm.

**Fig. (3):**
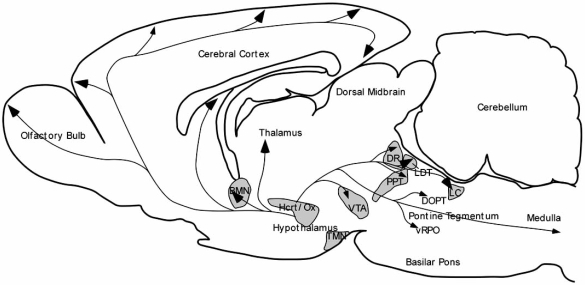
Sagittal scheme of the rat brain illustrating hypocretinergic influences on the cerebral cortex and wakefulness-promoting structures. Hypocretin/orexin (Hcrt/Orx) hypothalamic neurons send axons to both the cerebral cortex and neurochemically-specific neuronal groups projecting to the cortex, which are most involved in wakeulness maintenance and cortical activation. These groups are the noradrenergic locus coeruleus (LC), serotonergic dorsal raphe nucleus (RDo), cholinergic laterodorsal tegmental (LDT) and peduculopontine tegmental (PPT) nuclei, dopaminergic ventral tegmental area (VTA), histaminergic tuberomammilary nucleus (TMN) and cholinergic basal forebrain (BF) In the pontine tegmentum, Hcrt/Orx axons reach DOPT, where Hcrt/Orx enhance wakeulness, and also vRPO, where Hcrt/Orx suppress REM. Figure modified from [19].

The Hcrt/Orx neurons in the rat are restricted to the tuberal region of the hypothalamus, particularly the perifornical region (PeF) and the lateral hypothalamic area (LHA) [18,75]. In the cat, Hcrt/Orx neurons are also concentrated in the same tuberal region, but extend widely to other hypothalamic areas [89,106] (Fig. **2**). Hcrt/Orxergic neurons are variable in size (diameter of cell body of 15–40 μm) and shape (spherical, fusiform, multipolar) [16,17,63], and they have been assumed to number from 1,100 to 3,400 in the whole rat brain [34,69]. The human LHA has been estimated to hold about 50,000-80,000 Hcrt/Orx neurons [59]. Hcrt/ Orx axons are very heterogeneous in morphology; they can be either thick and very varicose or thin and slightly varicose [69]. Although Hcrt/Orx neurons are scarce, they have a profuse projection system to numerous brain regions involved in arousal and cortical activation and in sleep-wakefulness cycle regulation. Among the main structures innervated by Hcrt/Orx neurons are the hypothalamus itself, the locus coeruleus (LC), the dorsal raphe nucleus (DR), and the cerebral cortex [50,53]. Hcrt/Orx neurons also innervate the brainstem reticular formation, including the REM sleep inducing region located in the ventral portion of the oral pontine reticular nucleus (vRPO) [66] (Fig. **3**).

## HYPOCRETIN/OREXIN RECEPTORS

Two Hcrt/Orx receptors (Hcrt/Orx1R and Hcrt/Orx2R) have been described. They show 64% amino acid identity and their structure is similar to most other peptidergic receptors, to which they show an approximately 25–35% amino acid identity [75,76]. The amino acid homology between human and rat Hcrt/Orx receptors is 94% for Hcrt/Orx1R and 95% for Hcrt/Orx2R. The respective affinities (expressed as EC50, the concentration of ligand needed to elicit half-maximum receptor response) of Hcrt/Orx1 and 2 for Hcrt/Orx1R are 30 nM and 2500 nM. However, Hcrt/Orx1 and 2 have affinities of 34 nM and 60 nM, respectively, for Hcrt/Orx2R [75]. This indicates that Hcrt/Orx2R is a nonselective, high-affinity receptor for both Hcrt/Orx neuropeptides, whereas Hcrt/Orx1R is selective for Hcrt/Orx1 alone. Hcrt/Orx receptors are highly specific for Hcrt/Orx neuropeptides; neuropeptide Y, secretin, α-melanocortin, and other neuropeptides do not activate Hcrt/Orx receptors [37,83] (Fig. **1**).

Hcrt/Orx1R couple exclusively to the Gq/11 subclass of heterotrimeric G proteins, whereas Hcrt/Orx2R can couple to Gq/11 or Gi/o proteins. Signaling through the Gq pathway results in nonselective cation channel activation leading to cellular depolarization, while Gi/o signaling activates inwardly-rectifying K^+^ channels and leads to cellular hyperpolarization. Thus it is thought that Hcrt/Orx1R-mediated signaling is excitatory through the Gq/11-mediated stimulation of phospholipase C, while Hcrt/Orx2R-mediated signaling can be either excitatory (when coupled to Gq/11) or inhibitory through adenylate cyclase inhibition (when coupled to Gi/o), depending on the postsynaptic neurons [45].

The receptors are distinct gene products (*hcrt*-*r*1 and *hcrt*-*r*2) that show an apparently segregated form of mRNA expression in the rat. For example, *hcrt*-*r*1 mRNA is present in the LC, whereas *hcrt*-*r*2 mRNA is barely detectable [90]. Rat *Hcrt/Orx1R* and Hcrt/Orx*2R* mRNAs are detected on postnatal day 1 and embryonic day 18, respectively, suggesting the presence of Hcrt/Orx receptors at an early stage in hypothalamic development [93].

The mRNA distribution of Hcrt/Orx1R and of Hcrt/ Orx2R have been mapped in the complete adult rat brain. *Hcrt/Orx1R* mRNA was located in the prefrontal and infralimbic cortex, hippocampus, paraventricular thalamic nucleus, ventromedial hypothalamic nucleus, DR, and LC. *Hcrt/Orx2R* mRNA was detected in cerebral cortex, basal forebrain (BF) cholinergic nuclei, hippocampus, midline and intralaminar thalamus, raphe nuclei, and hypothalamic nuclei such as the tuberomammillary nucleus (TMN), dorsomedial, paraventricular, and ventral premammillary nuclei [30].

The distribution of Hcrt/Orx receptors is on the whole consistent with the location of the Hcrt/Orx axons and Hcrt/ OrxR mRNA-expressing neurons. Thus, the distribution patterns of Hcrt/Orx1R and Hcrt/Orx2R coincide in some regions but are distinct and complementary in some others. This suggests different physiological roles for each receptor subtype. Most of the noradrenergic LC neurons and cholinergic neurons in the pedunculopontine (PPT) and laterodorsal tegmental (LDT) nuclei express *Hcrt*-*r*1 mRNA and Hcrt/Orx1R. In contrast, serotonergic DR neurons and dopaminergic ventral tegmental area (VTA) neurons express *Hcrt*-*r*1 mRNA and Hcrt/Orx1R and *Hcrt*-*r*2 mRNA and Hcrt/Orx2R in a more balanced manner. In the forebrain, the histaminergic TMN exclusively expresses *Hcrt*-*r*2 mRNA and Hcrt/Orx2R.

*OX1R* mRNA has also been detected in structures other than CNS, such as the human adrenal zona fasciculata-reticularis and adrenal medulla, which show very low levels of *OX2R* mRNA [55]. However, Jöhren and coworkers [40] demonstrated that the amount of *OX1R* mRNA in the pituitary gland and of *OX2R* mRNAs in adrenal glands is higher in male than in female rats. These results suggest a sexually dimorphic role for Hcrt/Orx neuropeptides in peripheral organs that is still poorly defined.

## ELECTROPHYSIOLOGICAL EFFECTS

Hcrt/Orx peptides have been shown to exert excitatory actions on noradrenergic LC neurons, histaminergic TMN neurons, and cholinergic mesopontine and BF neurons [11,13,21,22,32,38,39,99].

Whole-cell patch clamp recordings in slices from neurons of the rat LHA, superficial dorsal horn or laterodorsal tegmentum demonstrated an increase in the frequency of spontaneous and evoked excitatory or inhibitory postsynaptic potentials (EPSPs and IPSPs, respectively) when Hcrt/Orx was administered [13,31,92,93]. Also, cortical neurons in layer VI are activated by Hcrt/Orx through the closure of a potassium conductance [5].

Moreover, Hcrt/Orx, acting on Hcrt/OrxR2 receptors, has been reported to depolarize neurons and increase their excitability either by activating an inward current [22,98] or by inhibiting an outward current [4]. The former occurs in TMN [22] and hippocampal [98] neurons and involves the activation of a Na^+^/Ca^2+^ exchange current. Moreover, activation of postsynaptic Hcrt/OrxR2 receptors also stimulates a Na^+^/ Ca^2+^ exchange current in arcuate Type-C GABAergic neurons, thereby producing membrane depolarization and an increased firing rate. This effect is dependent on an increase in cytosolic Ca^2+^ concentration, which is probably derived from intracellular stores [14].

Van den Pol and colleagues [93]. have studied the second messenger system involved in Hcrt/Orx signaling. Both types of Hcrt/Orx increase Ca^2+^ influx in medial and lateral hypothalamic neurons, as measured by fura-2 imaging, in about one third of hypothalamic neurons, probably by opening a plasmatic membrane Ca^2+^ channel. Hcrt/Orx responses are completely blocked by the PKC-specific inhibitor bisindolylmaleide, suggesting that Hcrt/Orx may work *via* Gq-activated PKC, resulting in Ca^2+^ channel phosphorylation that has been reported to increase Ca^2+ ^conductance [52]. More recent studies have shown that Hcrt/Orx may be linked to the adenyl cyclase pathway [55], probably *via* an interaction between Hcrt/Orx-2 neuropeptides and Gi proteins [41,71].

## HYPOCRETINS/OREXINS AND ENERGY HOMEOSTASIS

The hypothalamus has long been implicated in the regulation of food intake, body mass, body temperature and energy balance. The LHA would be responsible for the initiation of food intake, while the basomedial hypothalamic nuclei are associated with the cessation of food intake [7,8]. Moreover, Hcrt/Orx1 also increases food intake in satiated rats when infused intracerebroventricularly [102,103]. Furthermore, intraperitoneal injection of the selective Hcrt/ Orx1R antagonist (SB-334867-A) significantly reduced food intake and increased resting behavior in rats [35,73].

The molecular bases of food intake control are the appetite-stimulating (orexigenic) neuropeptides, such as melanin-concentrating hormone (MCH) [94], galanin [56], and dynorphin [95], which have been reported in the LHA neurons. In addition to food intake, Hcrt/Orx neuropeptides have also been implicated in the regulation of drinking behavior [46].

The Hcrt/Orx system is activated in situations in which little food is available, since 48-h fasting increases *prepro-Hcrt/Orx mRNA* levels in rats [75]. Insulin-induced hypoglycemia activates of Hcrt/Orx neurons, as determined by immunohistochemical staining against Fos protein [61]. Fasting in humans (ten nonobese females) results in an increase in plasma Hcrt/Orx1 paralleled by a reduction in plasma leptin levels [43].

Consequently, data indicate that Hcrt/Orx neurons are involved in an appetite regulatory circuit that includes the circulating hormone leptin, which is secreted by adipocytes according to total body adipose mass. The actions of leptin are partly mediated by the LHA, where it decreases the firing rate of both glucose-sensitive and glucose-insensitive neurons. In contrast, Hcrt/Orx1 increases the activity of glucose-sensitive neurons [79]. Patch-clamp measurements in isolated Hcrt/Orx neurons indicate that leptin, as well as high extracellular glucose levels, can directly decrease the neuronal firing rate and intracellular Ca^2+^ concentrations [62]. Exogenously administered Hcrt/Orx neuropeptides themselves also reduce the firing rate of these neurons. It is therefore likely that some of the leptin-sensitive and glucose-sensitive neurons in the LHA described by Shiraishi and coworkers [79] are in fact Hcrt/Orx neurons, and that these cells express inhibitory Hcrt/Orx autoreceptors.

It has also been pointed out that Hcrt/Orx can play a role in the control of body temperature. Anatomical evidences have demonstrated polysynaptic connections to thermogenic sites, such as the brown adipose tissue, from Hcrt/Orx neurons in the lateral hypothalamus suggesting the possibility that these neurons represent the anatomical substrate for two independent components for energy homeostasis, feeding and thermogenesis [67,68]. On the other hand, intracerebroventricular injections of Hcrt1/OrxA in mice neither increased the metabolic rate nor modified the body temperature, while the receptor antagonist SB-334867-A injected intraperitoneally acts as a thermogenic agent producing a significant increase in energy expenditure [36,51]. These two different effects can be due to that the antagonist has a direct effect on peripheral thermogenic sites although orexin release at these sites has not been demonstrated [36]. Since a close relationship between body temperature cycle and sleep-wakefulness cycle has been widely demonstrated (se for review [29]), it could be possible that Hcrt/Orx participate in the mediation of this relationship.

## HYPOCRETINS/OREXINS AND SLEEP-WAKEFULNESS CYCLE

The stages that characterize the sleep-wakefulness cycle are distinguished by different electrophysiological patterns in the electroencephalogram (EEG) and in other bioelectrical signals. Wakefulness is characterized by low-amplitude and fast EEG, while slow wave sleep (non-REM sleep) by high amplitude and slow EEG waves. This pattern develops further into high-frequency EEG waves that define the stage of REM sleep. Switching among these states is controlled in part by the activities of hypothalamic neurons and several areas located in the brainstem.

The Hcrt/Orx neuropeptides have been implicated in the control of the sleep-wakefulness cycle. Since the Hcrt/Orx neuropeptides were discovered, much data has been collected about their ability to enhance wakefulness and cortical EEG activation. Intracerebroventricular infusion of Hcrt/Orx1 produces an increase in wakefulness at the expenses of non-REM sleep and a remarkable decrease in REM sleep [32]. Moreover, most of the neurons within the PeF area, including the Hcrt/Orx neurons, increase their firing rate during alert wakefulness and decrease their activity during slow wave sleep and REM sleep in absence of twitches [2,44, 48,58]. However, Torterolo and coworkers [87] reported that significant *c-fos* expression in Hcrt/Orx-containing cells was detected during both active wakefulness and the carbachol induced REM sleep-like state. They found that 79% of the total number of hypocretinergic neurons detected were active during active wakefulness, approximately 34% of them were active during carbachol induced REM sleep, and only 2% were active during quiet wakefulness. Moreover, Kiyashchenko and coworkers [42] described maximal Hcrt-1 release in the hypothalamus and basal forebrain during both REM sleep and active wakefulness and minimal release during slow wave sleep. Thus, it is possible that the level of Hcrt/Orx1 may dependent on the intensity of motor system activation (see below) since central motor systems reach discharge levels equal to or greater than those of active waking during REM sleep and have minimal discharge during slow wave sleep [80,81].

The implication of Hcrt/Orx in sleep-wakefulness control is certainly the consequence of the existence of strong anatomical connections from Hcrt/Ox neurons to the major areas responsible for the generation of the different sleep-wakefulness states [24,25,63,69,107] (Fig.**3**). Hcrt/Orx neuropeptides excite DR, LC, TMN, LDT and PPT nuclei, as well as BF cholinergic neurons, by activating postsynaptic receptors in these neurons [11,21,23,32,39]. These “wake-active” nuclei are implicated in maintaining wakefulness. Accordingly, Hcrt/Ox neuropeptides promote wakefulness when administered in these regions [11,24,85,99]. Monoaminergic neurons in these nuclei are most active during wakefulness, slow down during non-REM sleep, and nearly cease firing during REM sleep, probably due to a decrease of the excitatory Hcrt/Orx inputs.

In relation with the control of REM sleep generation, Hcrt/Orx projections and receptors have been identified in cholinoceptive areas of the pontine reticular formation involved in REM generation and control of REM-polygraphic signs [30,53,97,107]. Furthermore, Hcrt/Orx enhances acetylcholine and GABA release in these areas [6,96]. However, altering Hcrt/Orx neurotransmission in the pontine tegmentum has led to conflicting results in behaving animals. Some studies have reported a facilitation of REM sleep after Hcrt/Orx increase in the pontine tegmentum [99-101] but others groups have reported a Hcrt/Orx inhibitory action on REM sleep [10,84].

These discrepancies may be the result of the different cellular actions produced by Hcrt/Orx at the level of the dorsal oral pontine tegmentum (DOPT) and in the ventral part of the oral pontine reticular nucleus (vRPO), which is implicated in REM sleep generation [27,28,60,72], Hcrt/Orx in DOPT was recently found to produce excitatory electrophysiological responses in both cholinergic and noradrenergic cells [12]. In contrast, we have demonstrated that iontophoretic application of Hcrt/Orx through a barrel micropipette in the vRPO induces inhibition by activation of GABA_A_ receptors because is blocked by application of the GABA_A_ antagonist bicuculline [66]. There is a specific Hcrt/Orx projection from the PeF area to the vRPO [66]. Therefore, the PeF area might control REM generation through a hypocretinergic projection that would activate GABAergic mechanisms.

**Fig. (4):**
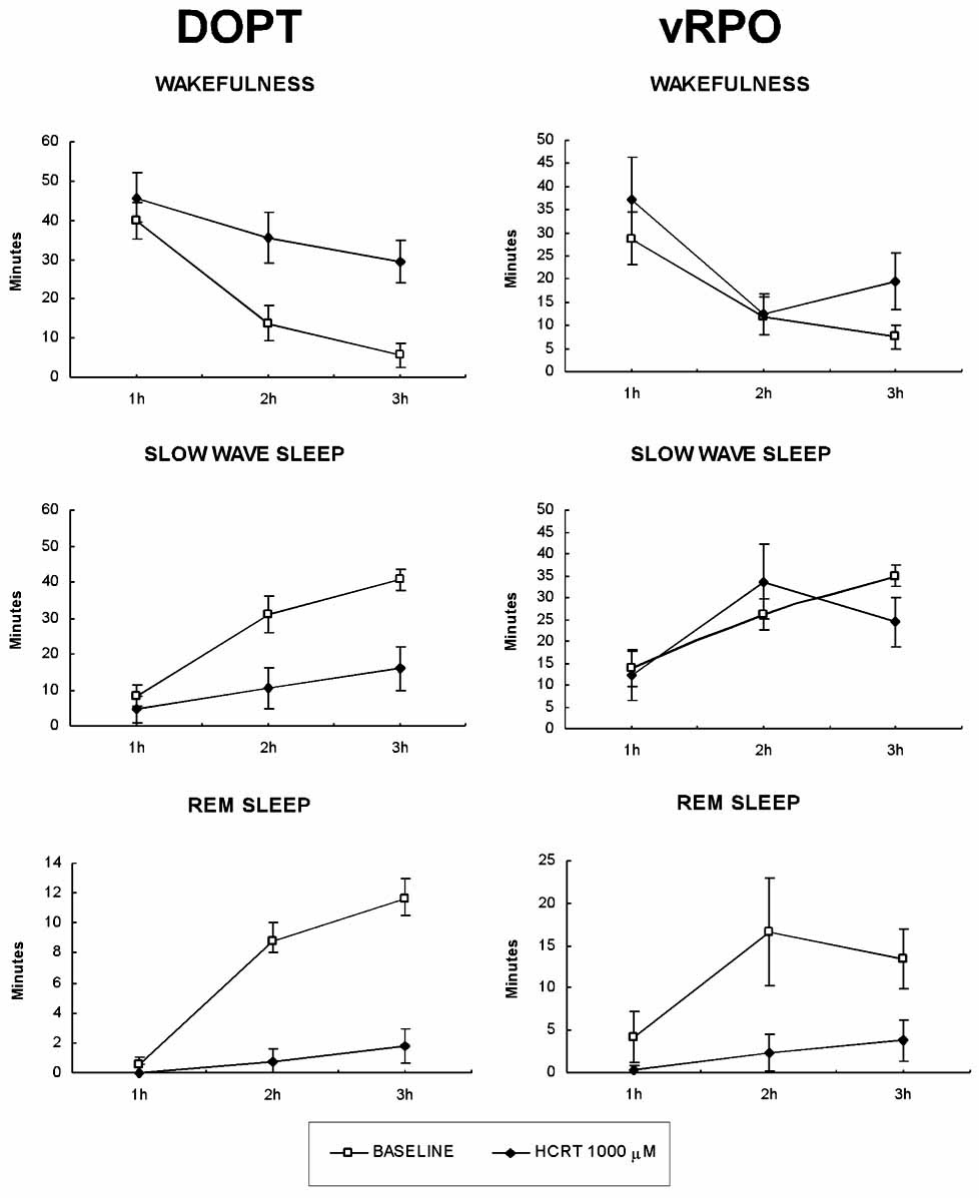
Mean time spent ± SEM of the sleep-wakefulness cycle states by animals with Hcrt-1 microinjections in either the dorsal oral pontine tegmentum (DOPT) or the ventral oral pontine tegmentum (vRPO) in each of the first 3 h of polygraphic recordings in baseline and after Hcrt-1 1000 mM dose experiments. *Statistically significant difference in comparison with baseline. Post hoc analyses (Fisher's test, P < 0.05).

Recent experiments in our laboratory have shown that Hcrt/Orx neuropeptides have a wake-promoting and sleep-suppressing actions when acting in the DOPT and a direct and exclusive inhibition of REM sleep when acting in the vRPO [60] (Fig.**4**). Also a defacilitating action on REM sleep could be secondarily produced by the wake-promoting and sleep-suppressing actions of Hcrt/Orx in other pontine areas such as the principal LC and LDT nuclei [11,99]. The loss of Hcrt/Orx signaling in narcolepsy disease would impair these actions and could remove the defacilitating/inhibiting actions on REM generation of the Hcrt/Orx signal in these pontine regions during wakefulness; consequently, patients would fall directly into REM while still in a wakefulness period (see below).

Hcrt/Orx neurons may be also involved in motor activity. Hcrt/Orx cells discharge during active waking, when postural muscle tone is high in association with movements, decrease discharge during quiet waking in the absence of movements, and virtually cease firing during sleep, when postural muscle tone is low or absent [2, 48, 58]. However, Hcrt/Orx-containing neurons are also activated during carbachol-induced REM sleep with muscular twitches [88]. The relationship between hypocretinergic system activation and motor activation is reinforced by decrease in Hcr/Orx1 levels in CSF of rats after long-term immobilization and its increased levels after short-term forced swimming [54,86]. The peptide concentration in dialysates from the hypothalamus was significantly higher during active waking than during slow-wave sleep [42]. Moreover, systemic, intracerebroventricular, and intraparenchymal injection of Hcrt/Orx increases motor activity [42,86].

In agreement with a putative role for the hypocretinergic system in motor functions, Hcrt/Orx terminals have been found in the ventral horn where motoneuron cell bodies are located [91]. In addition, application of hypocretin depolarizes lumbar motoneurons by means of presynaptic and postsynaptic mechanisms that result in the facilitation of their discharges [104]. These authors propose that this action of Hcrt on motor output is important in the physiological regulation of motor activity in situations that involve certain hypothalamus-driven behaviors.

Another question of interest is the mechanism for circadian regulation of Hcrt/Orx neurons. As mentioned above, Hcrt/Orx neuron activity follows a circadian rhythm as demonstrated by both Fos-immunostaining [26] and Hcrt/Orx peptide levels measured in the rat cisterna magna [105]. Hcrt/Orx neurons in rats and humans were recently shown to be directly innervated by neurons of the suprachiasmatic nucleus, a structure that is responsible for regulation of circadian processes [1]. Hcrt/Orx neurons may therefore be a relay station for circadian sleep/wake control by the suprachiasmatic nucleus.

## HYPOCRETINS/OREXINS AND NARCOLEPSY

Idiopathic narcolepsy is more frequent than commonly thought, having approximate prevalences 1 in 1,000–2,000 in the United States [82] and 1 in 600 in Japan [57]. This neurological disorder is characterized by a primary disturbance in sleep-wakefulness organization. The onset of narcolepsy most often occurs during adolescence and the symptoms gradually reach a certain severity within several years, after which patient condition neither worsens nor improves.

Narcoleptic patients suffer from severe daytime hypersomnolence, combined with night time insomnia and sleep fragmentation, which produces a constant feeling of tiredness in these subjects. In healthy human subjects the latency for REM sleep after the onset of non-REM sleep is around 90–100 min. In contrast, in narcoleptic patients, REM sleep latency is frequently shortened to less than 15 min, sometimes being so short that even direct transitions from wakefulness to REM sleep occur, something which can understandably cause embarrassing and even dangerous situations. This “sleep-onset REM period” is regarded as the diagnostic indication for narcolepsy.

However, the most striking feature of the disease is cataplexy, a sudden bilateral loss of skeletal muscle tone during wakefulness; it is most often triggered by a strong positive swing of emotion such as laughter (a trigger in 80% of cases) [3]. Cataplectic attacks normally last from a few seconds to a few minutes and range in severity from slurred speech, head dropping, and knee jerking to complete collapse to the floor despite maintained consciousness [3]. All these clinical symptoms suggest that narcolepsy is a dysfunction of vigilance state boundary control, in which the fundamental pathophysiology involves an abnormal and premature intrusion of REM sleep into the state of wakefulness.

Current pharmacological treatment of narcolepsy is based on two approaches, although a host of different therapies are in use [64]. Excessive daytime sleepiness is currently treated with either amphetamine-like stimulants or the stimulant modafinil, both of which increase the catecholaminergic tone. Amphetamines increase catecholamine release and also reduce catecholamine uptake by inhibiting monoamine transporters, however they have considerable sympathetic side effects. Modafinil is structurally unrelated to amphetamines and presently constitutes a better first-line treatment for excessive daytime sleepiness and sleep attacks. Although the mechanism of action of modafinil is not yet fully understood, it is thought to consist mainly in inhibition of the dopamine transporter. Interestingly, administration of modafinil or amphetamine-like stimulants to mice increases Fos-expression in Hcrt/Orx neurons of the hypothalamus [15] or of the TMN [77]. Since both amphetamines and modafinil also enhance wakefulness in Hcrt/Orx-deficient narcoleptic subjects, it appears that their sites of action are largely independent of the Hcrt/Orx system, and their advantageous actions in narcolepsy would be purely symptomatic.

Despite promotion of wakefulness, these stimulants do not improve other REM sleep-related narcolepsy symptoms. For the treatment of cataplexy, tricyclic antidepressants such as imipramine, protryptiline, and clomipramine have been commonly used and are still widely prescribed. These drugs act by blocking reuptake of noradrenaline and serotonin, and they have considerable anticholinergic side effects [9]. The newer antidepressants, such as fluoxetine, are clinically less effective, although they have significantly less side effects. Sodium oxybate is, at present, the first-line treatment for cataplexy. It is the sodium salt of the natural neurotransmitter gamma-hydroxybutyric acid, and it binds to its own receptors at physiologic concentrations; however, when used at higher pharmacological concentrations, sodium oxybate acts mainly through GABA_B_ receptors.

Animal models of human narcolepsy consist in modifications of Hcrt/Orx receptors [49] or absence of these peptides [15]. Hcrt/Orx knockout mice display a severe narcolepsy-like phenotype [33]. This is also evident in double receptor knockout (Hcrt/Orx1R- and Hcrt/Orx2R-null) mice. In contrast, knockout mice for either Hcrt/Orx1R or Hcrt/Orx2R show phenotypes that is somewhat different. Hcrt/Orx1R deficient mice only exhibit slightly increased sleep fragmentation and lack evident behavioral abnormalities. Hcrt/Orx2R knockout mice also show a mild narcoleptic phenotype, in which fragmentation of sleep is present but abnormalities of REM sleep, such as direct transitions from wakefulness to REM sleep, are either absent or much less frequent than in double-null animals. These data suggest that Hcrt/Orx2R is critical for normal regulation of wakefulness/non-REM transitions, whereas the intense deregulation of REM sleep control present in the narcoleptic syndrome relies on signaling disruption through both Hcrt/Orx1R and Hcrt/Orx2R.

Nowadays it is assumed that narcolepsy is the direct consequence of Hcrt/Orx neuron degeneration, and therefore indicates widespread Hcrt/Orx hypofunction. There are different reasons to link Hcrt/Orx and human narcolepsy. Narcoleptic patients have fewer Hcrt/Orx neurons in the posterolateral hypothalamus than control subjects [70,86], and their cerebrospinal fluid shows lower or untraceable Hcrt/Orx levels [65]. Moreover, gliosis has been reported in the perifornical area in some narcoleptic patients [70,86]. All these observations, together with the well known association between narcolepsy and specific antigens of the major histocompatibility system (HLA), suggest that an autoimmune process might be the triggering factor initiating hypothalamic Hcrt/Orx neuron degeneration in narcolepsy. The astrocytic marker GFAP (glial fibrillary acidic protein) for gliosis seems to be present in a few narcoleptic patients, and might be found in more since the analyzed tissue had been stored for a long time and could have lost immunoreactivity [86]. Although Hcrt/Orx neuronal degeneration is the most accepted hypothesis for human narcolepsy, other possible causes, including defects in the synthesis of Hcrt/Orx or their receptors cannot be rejected. Hereditary canine narcolepsy caused by a mutation in *hcrt2R/ox2R* [49] or rodent models of narcolepsy due to deletion of the *Hcrt/Orx* gene [15] have been well documented.

At the present time, Hcrt/Orx neuropeptides are considered to be neuromodulators that enhance the waking state through increasing the activity of several neuronal populations; they also inhibit REM sleep by acting on the vRPO (see above). Impairment of the Hcrt/Orx neuron projection system or actions would provoke, on one hand, hypoactivity of the ascending activating systems, and, on the other hand, disinhibition of the vRPO and REM sleep triggering. This hypothesis could explain the great number of transitions between wakefulness and sleep, REM sleep fragmentation and hypersomnia present in narcoleptic patients.

## CONCLUSIONS

The Hcrt/Orx neuropeptide system has proven to be a novel mechanism by which the brain regulates arousal and sleep/wake states. Also, these neuropeptides contribute to regulation of energy homeostasis. The link between narcolepsy and Hcrt/Orx deficiency in animals and humans has provided a better understanding of sleep-wakefulness regulation and the cause of narcolepsy. Different studies clearly demonstrate that Hcrt/Orx neuropeptides favored the activity of neurons implicated in wakefulness generation while at the same time, they inhibit neurons involved in REM sleep generation.

Discovery of the pathogenic mechanisms that underlie the loss of Hcrt/Orx neurons in humans will constitute a crucial boost for narcolepsy research in the future. That information is essential for the prevention and treatment of this disease.
